# Computer Game-Based Telerehabilitation Platform Targeting Manual Dexterity: Exercise Is Fun. “You Are Kidding—Right?”

**DOI:** 10.3390/s21175766

**Published:** 2021-08-27

**Authors:** Sanjay Tejraj Parmar, Anuprita Kanitkar, Nariman Sepehri, Satish Bhairannawar, Tony Szturm

**Affiliations:** 1 SDM College of Physiotherapy, SDM University, Dharwad 580009, Karnataka, India; 2 College of Rehabilitation Science, University of Manitoba, Winnipeg, MB R3T 5V6, Canada; anuprita.kan@gmail.com; 3 Department of Mechanical Engineering, Price Faculty of Engineering, University of Manitoba, Winnipeg, MB R3T 5V6, Canada; nariman.sepehri@umanitoba.ca; 4 Department of Electronics and Communication Engineering, SDM College of Engineering and Technology, Dharwad 580009, Karnataka, India; satishbhairannawar@gmail.com

**Keywords:** cerebral palsy, stroke, manual dexterity, upper extremity rehabilitation, computer game-based rehabilitation, repetitive task practice

## Abstract

There is a need for innovation to improve the engagement and accessibility of rehabilitation programs for children and adults with upper extremity motor impairments due to neurodevelopmental disorders, acquired brain injuries, or spinal cord injuries. For this purpose, a computer game-based telerehabilitation platform (GTP) was developed to address this need. Through the application of a miniature inertial-based computer mouse and the wide variety of commercial computer games, the developed GTP can provide engaging task-specific exercises for the rehabilitation of manual dexterity (object handling and manipulation). A purpose-built repetitive task practice software (RTP) was also developed to gather event data and synchronize it with patient movements during gameplays. This provides automated monitoring and quantification of patients’ motor skills, while they practice a range of game-based exercises with their hand and/or arm. The GTP would initially be used in a supervised clinical setting followed by a transition to function at home and be monitored by clinician specialists. Clinical support for home and rural communities, with protocols that can be easily updated, will help increase accessibility to targeted and personalized solutions for patients and achieve the desired training effect.

## 1. Introduction

Many children and adults with neuromuscular impairments affecting the upper extremity have deficits in fine motor skills [1,2,3,4,5]. Manual dexterity involving the handling and manipulating of objects is essential for daily life activities, social life and, play activities for children. These activities require manipulation of objects with a wide range of physical properties and functional demands which often require a high degree of precision. Therapy programs designed to improve manual dexterity, eye-hand coordination, and other related visual-spatial processing skills must strive to maximize neurodevelopmental capacities and prevent secondary disabilities.

Constraint-induced movement therapy (CIMT) [6,7,8,9], and hand arm bimanual intensive therapy (HABIT) [10,11] are promising rehabilitation programs for the restoration of hand-arm function. These treatment approaches stress that functional recovery reflects learning achieved through generating real experiences, applying focused attention, simulating close-to-normal movements and repetition [12,13,14]. Supervised task-specific therapies are preferred. They are, however, challenging to deliver due to the early discharge of patients, increasing demands for rehabilitation services by an aging population, as well as financial and travel distance pressures [15]. Unforeseen situations (e.g., physical distancing imposed by the COVID-19 pandemic) can further complicate this process. To overcome these challenges, we need feasible and cost-effective methods to increase accessibility. Ideally, effective therapy programs should be made available in patient homes [16,17,18,19].

In addition to increasing accessibility, there is a need to improve compliance of exercise regimes. An emerging approach to engage clients in therapy is to incorporate computer games [20,21,22]. Interactive activities in computer games can provide a behavioral, ecologically valid, and adaptive environment. In addition, they can provide immediate feedback as well as electronic records to assess and assist in the progression of interventions. Several gaming systems have been used in rehabilitation of the upper extremity. These include, commercial entertainment gaming systems such as the Wii [23], Kinect [24], Leap’s motion sensor [25], and some use robotic manipulandam [26] or a sensor-equipped glove [27]. These gaming systems can, in real-time, detect arm segment motions or finger motions. The motion signals are used to control the position and motion of virtual avatars/objects, or to control a game paddle for play. However, these systems are limited because they do not target object handling and manipulation. Therefore, there is no consideration of sensory information required to maintain object stability during movement, or to detect and minimize slip of the moving objects [28,29]. It is crucial to create experiences that improve the brain’s ability to learn, and for manual dexterity this is best achieved by performing precise object manipulation tasks through guided and assisted repetition. Some game-based rehabilitation systems use a handle such as the game controller. The handle is grasped and moved using pronation-supination or elbow and shoulder motion [30]. This rehab gaming system involves a limited number of object manipulation tasks that can be coupled with computer games. In addition, it includes only a few custom games suitable for children and adults with motor impairments. There is a large number of inexpensive and readily available common and modern commercial games that are engaging, therapeutic, and can be played with a computer mouse or an equivalent [31,32,33]. Another rehabilitation system uses an iPhone, fitted to 3D printed objects (e.g., coffee mugs, bowls). The motions of these objects are captured to interact with several computer activities, including moving a virtual object from one location to another [34].

Based on the above considerations a low-cost computer game-based telerehabilitation platform (GTP) was developed that combines fine manipulation or gross movement exercises with game activities appropriate for young children with neurodevelopmental disorders [35,36], and adults with acquired brain and spinal cord injuries [37]. Finger-hand function is targeted to extend the utility beyond gross reaching or transport movements, since the ability to perform manual dexterity skills with hands is very much an integral part of everyday life.

The GTP organizes space of game interactions into two interrelated categories, physical space, and digital space.

## 2. Physical Space

**Figure 1:**
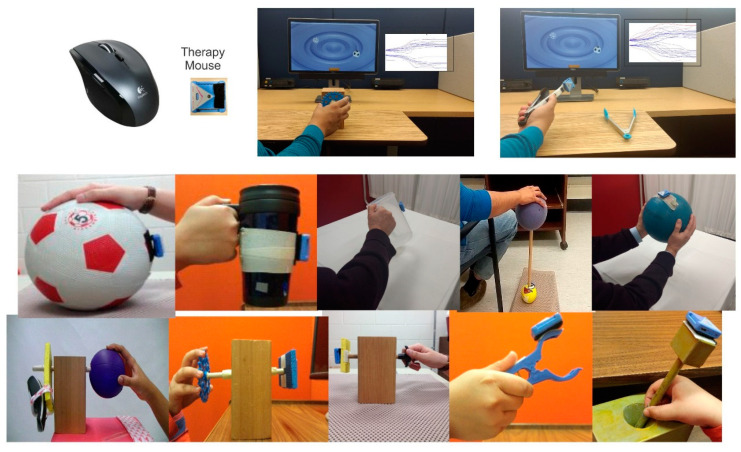
Components of game-based telerehabilitation platform (GTP). Top left panel shows miniature motion mouse (therapy mouse) next to a typical optical mouse. The motion mouse is attached with Velcro to various objects and is used to play computer video games, illustrated in top right panels.

A Miniature, wireless plug-n-play inertial-based (IB) computer mouse is used to directly link physical movements with interactive computer games. As shown in Figure 1, by simply attaching the IB mouse to an object, the object becomes a computer mouse. Therefore, the natural manipulation of object can be used to control the motion of the computer cursor or game paddle/sprite. Because the mouse can be easily attached with Velcro to any objects, this approach provides a highly flexible and personalized clinical or home-based treatment tool. Multiple objects, utensils, tools or toys with varied sizes, shapes, weights, surface properties and functional demands for precision can easily be instrumented with the miniature motion mouse and used for assessment and to practice a variety of gross or fine motor and psychomotor skills. Exercise objects can be chosen to be handled with a two-finger grip, three finger grip, whole hand grasp, or even both hands. Some object manipulation tasks require a combination of wrist, elbow, and shoulder motion.

Many common objects have ergonomic properties, which afford ease of manipulation. These are readily available to meet the needs of even the most involved and severely disabled clients with minimal range of movement and/or movement controls. This is a good starting point as it minimizes the need for expensive assistive robotic devices. For example, rolling a large cylinder object or sports ball using elbow-shoulder movement, which also requires finger/wrist extension, take advantage of the ergonomic properties of these common objects to amplify limited and small amounts of voluntary movement. It is easy to roll a cylinder or ball and the weight of the arm is supported by the object. With practice and improvement in an active range of motion and movement control, one can switch to objects having more demanding movement requirements and functional demands. A thorough knowledge about objects and their physical properties, functional and anatomical demands, will help to set specific treatment goals and to obtain objective outcome measures and monitor progression.

Because this method allows the handling and manipulation of objects, utensils, tools, and toys commonly used by adults and children in daily activities, the goals to attain a wide range of functional demands can easily be incorporated into personalized therapy. Thus, the approach focuses not only on body functions and structures as described by the World Health Organization International Classification of Function (ICF) but also can include goals and outcomes related to carrying out daily activities.

## 3. Digital Space

The IB mouse functions as a responsive USB plug-n-play computer mouse and this allows common and modern computer games to be used and enjoyed as part of a clinical and home rehabilitation program. Inclusion of a gaming element is intended to provide extra motivation for the patients in the form of a challenge and a more enjoyable means of encouraging them to follow tedious, repetitive movements that are often a part of the rehabilitation process. Specific therapeutic value can be derived from both the types of object manipulation tasks, as well as the choice of computer game. Different commercial games require different levels of movement amplitude, speeds, accuracy, and repetition, and offer sufficient diversity to appeal to a broad range of individual preferences. Updating and progressing the games and difficulty levels regularly, to sustain the challenge and provide new experiences, will facilitate the psychological feedback required to maintain interest and participation. Many commercially available games also involve multi-tasking. Hence, the tasks also engage/improve key attentional, perceptual, and cognitive skills. However, due to the lack of access to source codes and the typically coarse performance measures available from most commercial gaming packages, a repetitive task practice (RTP) software was developed and validated [38]. The RTP software gathers event data and synchronizes it with user movements during game plays [38]. This provides automated monitoring and quantification of players’ motor skills while they practice a range of game-based exercises with hands and arms; this way, assessment is embedded into treatment and telemonitoring. Several objective outcome measures are determined allowing clinicians to track both compliance and changes in function, so that they can design sustainable and improved individualized programs. Clinical support of home and remote outreach programs with protocols that can be easily updated will help create better-targeted and personalized solutions for patients and achieve the desired training effect.

The RTP game application includes two assessment modules used to standardize object manipulation tasks: (a) a cyclic tracking (CT) module and (b), a motor skill game (MSG) module [38].

## 4. Cyclic Tracking (CT) Module

**Figure 2:**
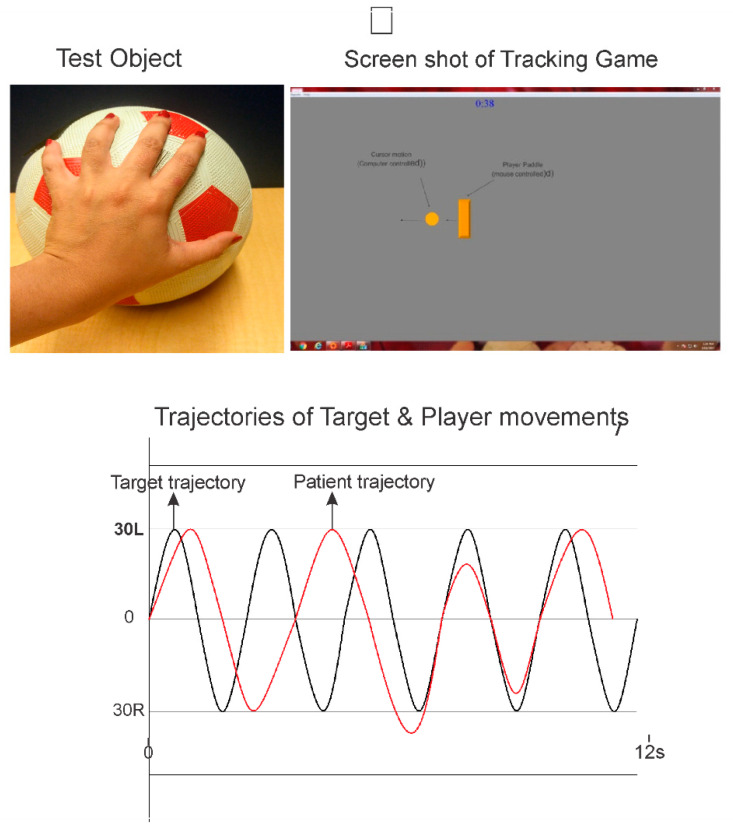
Illustration of an assessment using the cyclic tracking module. In this case, the RMD handle manipulation requires fine rotation using abduction-adduction and flexion-extension of the thumb and fingers. Top right panel shows a screenshot of the tracking task, goal is to track and overlap the game paddle (rectangle object with the moving circle object (computer controlled)). Bottom plots show typical movement trajectory of a healthy young adult performing the tracking task. It is played for 30 s to obtain several continuous tracking cycles for analysis.

A moving visual target on a computer display is used to guide and pace the client’s movements. As illustrated in Figure 2 the goal is to track and overlap the rectangular game paddle with the moving target circle object. The paddle is slated to the rotation of the object equipped with the IB mouse. The target is computer-controlled amplitude and frequency of which is configurable. The motion of the target can be horizontal (left to right) or vertical (top to bottom). The client’s tracking movements can be analyzed for movement errors and movement consistency. Figure 2 presents synchronous plots of the target motion (circle cursor) and that of the mouse equipped object rotation (rectangle cursor) for a cyclic tracking trial. The following performance measures are quantified:

1. Total and Average Residual Error (TRE): For each sampled data point, the difference in position (residual error) between the target position (circle) and the patient’s position (rectangle) is computed. Next, the total and average residual errors of all samples are determined;
2. Amplitude Variation (AV): First, the movement amplitude for each half cycle was determined. Next, the average movement amplitude and standard deviation of the movement cycles are computed. The coefficient of variation (COV) in the movement amplitude is computed as the standard deviation divided by the mean amplitude and expressed as a percentage of 100%.

Note the first two cycles are not included in the computation of either TRE or AV, as it often takes one or two cycles for the subject to begin the tracking task.

## 5. Motor Skill Game (MSG) Module

**Figure 3:**
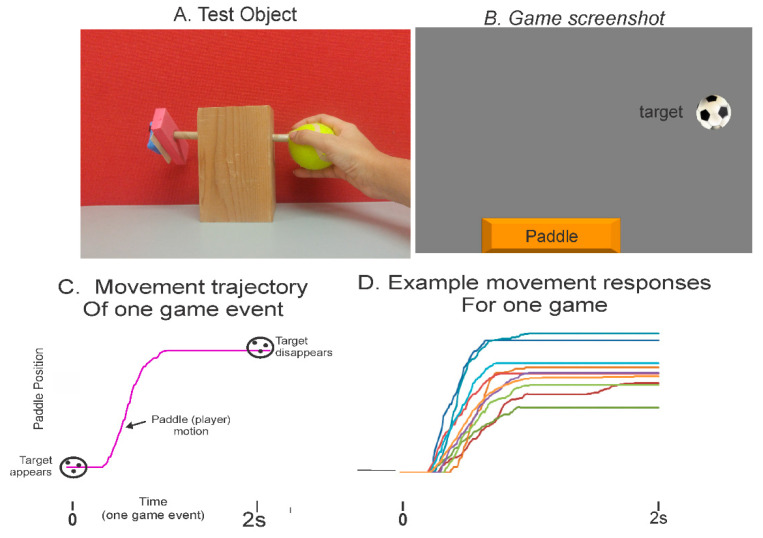
Assessment illustrations using the motor skill game (MSG) module. (**A**) A simple crafted object with motion mouse attached to the left end of wooden dowel by velcro. As seen, finger pinch grip is used to rotate the dowel. (**B**) A screenshot of game showing target object (computer controlled) and game paddle (slaved to rotation of the tennis ball using the motion mouse). (**C**) Trajectory of game paddle of one typical game event from target appearance (time zero) to target disappearance (time 2 s). (**D**) Overlay plots of the segmented and sorted game movement trajectories for a 60 s game trial.

This module requires the client to interact with various moving game objects. As illustrated in Figure 3, the game objects appear at random locations on the computer screen. The patient manipulates the object to control the motion of the game paddle that interacts with the moving game objects. For example, a typical game is played for 60 s, and the duration of each game event (from object appearance to its disappearance) can be set to 2 s. In this case, the patient makes 30 goal-directed game movement responses. These contextual game movement responses can be analyzed for success rate, response time, movement time, accuracy, and movement variation. In addition to target objects that are to be tracked and caught, there are distractor objects that must be avoided. Distractor objects can be turned off depending upon the required complexity of the game, or cognitive processing load. Target and distractor objects appear at random locations of the display and move to the opposite edge of the monitor. There are many difficulty levels ranging from very easy to difficult; therefore, it can accommodate individuals with all types of movement control. The following features and game elements are configurable:

1. Movement speed;
2. Movement precision (size of game object/game paddle);
3. Movement amplitude;
4. Addition of distractors, to evaluate interplay of motor and cognitive processing or dual-task interference effects;
5. Complexity of game trajectories (straight or diagonal).

A data logger records the following information:

1. The time of appearance and disappearance of each game object, which defines a game event;
2. The position coordinates (both cyclic tracking and motor skills) of player-controlled rectangle and game-controlled objects, required to determine movement context and precision.

The sampling rate is set at 100 Hz. Each of the samples is recorded in an output data file that is stored on the local computer or can be made available to upload to a content management system.

Figure 3C presents the typical plot of a patient’s game movement response. Figure 3D presents overlay plots of all game movement responses in each direction for one motor skill game trial, for a duration of 60 s. Several performance metrics are computed, which represent different features of the player’s performances; they include goal attainment, success rate, information processing and motor planning time, spatial aspects of movement precision and movement consistency as measured over multiple responses.

## 6. Reliability Testing

Test-retest reliability of the computerized upper extremity (CUE) assessment tool was evaluated in a group of 30 stroke patients [39], and 35 children with cerebral palsy [40]. Several object manipulation tasks were chosen to span a broad range of functional properties and anatomical requirements. Testing was evaluated on two occasions scheduled 1 week apart. All tasks required finger-thumb or hand palmer surface contacts, and involved various combinations of precision finger, wrist, elbow, and shoulder movements. For example, with reference to Figure 1, using a soccer ball, participants placed their open hand near the top surface to roll it forward and backwards using a combination of elbow extension-flexion and shoulder flexion-extension. When using a coffee mug, participants grasp the mug and moved it with concentric pronation and eccentric supination, while keeping a forearm supported on the tabletop. Utilizing a bimanual, participants held a large inflatable beach ball between both open hands and moved it up and down using elbow flexion-extension. Implementing fine rotation tasks, participants were asked to grasp a small diameter object (end of a wooden dowel) with the tips of their index finger and thumb and to rotate the wooden dowel. The CUE outcome measures included the success rate, movement onset time, and magnitude of movement errors. With few exceptions, the CUE assessment tool showed moderate to high test-retest reliability; interclass correlation coefficients ranging from 0.5 to 0.85. This was the case for both stroke patients [39] and children with cerebral palsy [40]. Relatively low minimal detectable change (MDC) scores of less than 20% of mean values, were observed for the stroke patients. However, the minimal detectible change values were notably large (30–70% of group mean values).

## 7. Clinical Studies

A mixed-method exploratory randomized controlled trial (RCT) was conducted to evaluate the disability, acceptance, and benefits of the GTP for children diagnosed with cerebral palsy (CP) [41] The objectives of the study were to explore parental views of the children’s experiences with the exercise program, and to provide an estimate of the treatment effect size that would direct a future full-scale RCT. The qualitative analysis is important for gaining knowledge from children’s experiences with the game-based exercise program regarding difficulties with the exercises and the technologies as well as the engagement and motivational value of the computer games [42].

Sixty-three children with cerebral palsy who meet the inclusion criteria were recruited for this single, blind randomized clinical trial with an active control arm [42]. The inclusion criteria were:

1. Gross Motor Function Classification System (GMFCS) levels I-III;
2. Manual Ability Classification System (MACS) levels I-III;
3. Modified Ashworth scale level of spasticity in finger and wrist extensors less than grade 2;
4. The pediatric version of the Mini Mental Status Evaluation (MMSE) score of 17 and above.

The following assessments were used to evaluate the treatment effects after a 16-week intervention program delivered three times per week: (a) Peabody Developmental Motor Scale-2 (PDMS-2) grasping and visual-motor integration (VMI) subtests; (b) CUE assessment of five object manipulation.

The experimental group (XG) received the GTP exercise program. This consisted of task practice of between six to eight object manipulation tasks coupled to several computer games. The exercise objects were common objects used in daily activities and play and represented a wide range of physical properties requiring different modes of manipulation and functional demands. The computer games were chosen based on the following requirements: (a) movement amplitude to move the game paddle, (b) and game speed and game precision. In addition, cognitive load was increased by selecting games with an increasing number of distractor objects. The list of commercial games that were used in this study is provided in Appendix A. The control group (CG) received a therapy program that consisted of task specific training similar to the tasks used in studies of modified CIMT [7,8] and HABIT [10,11].

Exercises were individualized for each child in both XG and CG according to their level of impairment and pre-set goals. The children with the guidance and assistance of a therapist practiced their respective exercises. New tasks and challenges were added as the child progressed.

The mean age and standard deviation of the control group were 7.2 and 2.1, respectively. They were 7.8 and 1.9, respectively, for the experimental group. There were three dropouts in the CG and none in the XG. All participants in both groups attended all 48 exercise sessions (three per week for 16 weeks). Most parents of the children participating in the study identified the focus on handling and manipulating objects as an important exercise feature of the GTP and that it included many different objects used in day-to-day life [42]. Parents from the XG reported that the exercises were challenging, yet engaging and, their children enjoyed playing the games. They felt that this gratification was important and improved children’s compliance with the therapy program. Previous studies that have compared the results of the use of computer games versus conventional therapy in terms of patient acceptability have observed similar results [2,43].

Both the XG and CG showed significant improvements post intervention with medium to large effect sizes in the PDMS-2 grasp and VMI tests scores, as well as the CUE assessment outcome measures [41]. Improvements in the PDMS-2 grasp and VMI scores, observed in the XG, were significantly greater than those in the CG. The GTP object manipulation tasks required precision, i.e., goal-directed movements based on visual feedback of the moving game objects. This is reflected by the improvements in performance of both the PDMS-2 visual-motor tasks and the CUE object manipulation tasks. The significant improvements observed in the fine motor tasks of the PDMS-2 occurred even though these tasks were not practiced during the intervention. For example, tasks such as drawing, coloring and managing to button a shirt.

## 8. Telerehabilitation Trial of Stroke Patients

The feasibility and benefits of a home exercise program using the GTP was examined in ten stroke patients [44]. Ten individuals who experienced a single stroke within a two-year period, with a mean age of 58 ± 12 years (four females and six males) participated in this study. Pre- and post-intervention assessments included the Wolf Motor Function Test (WMFT) and the CUE assessment of five object manipulation tasks. Each patient received four therapy sessions for training with the GTP. During these sessions, participants were taught a set of hand-arm exercises. Once the participants were familiar with their exercise program, the use of the IB mouse, and the computer games, they were asked to continue the program at home four times per week for 16 weeks. Several exercise objects and computer games were given to each participant for home use by the therapist. Regular weekly discussions with the participants by telephone provided feedback, guidance on progression, and switching to more challenging games and object manipulation tasks. In addition, two follow-up sessions were scheduled at 2 weeks and 8 weeks of the home program, where the exercises of the participants were re-evaluated and updated. Eight of the ten participants fully complied with the 16-week exercise program while two participants had difficulty with computer operations and did not complete the program. For the eight participants who completed the program, there was a substantial improvement pre- to post-intervention in the WMFT and in manual dexterity CUE assessment outcome measures.

## 9. Telerehabilitation Trial of Children with Cerebral Palsy

The feasibility and benefits of the GTP was recently conducted with six children diagnosed with cerebral palsy. Table 1 presents the demographic and clinical data of each child participant (P1–P6). This includes age, gender, GMFCS and MACS. The baseline scores of the PDMS-2 grasp and VMI subtests are presented in Table 2.

After the initial screening process, parents provided written informed consent, and ascents were obtained from the children. Ethical approval for the study was obtained from the Institutional Ethical Committees at SDM College of Medical Sciences and Hospital Ethics Committee in Dharwad, India and the Health Research Ethics Board of the University of Manitoba, Canada.

Each child and parent attended four 45 min therapy sessions. During these sessions, the child and parent were taught a set of hand-arm exercises. The starting exercise program was established based on their personal goals and the degree of their impairment. Typically, six to eight objects were selected for motor skill training of finger/wrist and a combination of elbow and shoulder motions, as well as bimanual tasks. A wide variety of games were played and reviewed during the initial four training sessions. Games were selected based on the following requirements: (a) movement amplitude to move the game paddle, (b) game speed and (c), game precision requirement. Many readily available and inexpensive computer games have therapeutic value. One good example is the commercial internet website, Big Fish Games [45]. It contains hundreds of arcade-style computer games in several genres with varying difficulty (speed, accuracy) levels. In addition to the movement requirements, the games used in this study, included several cognitive elements. Appendix A provides a list and brief description of example games used in this study. Once the children and the parent were familiar with their exercise program, the use of the IB mouse, and the computer games, they were asked to continue the program at home four 12 weeks, four times per week for 30 min sessions. Several exercise objects and computer games were given to each participant for home use by the therapist. Regular weekly discussions with the parents by telephone provided feedback, and guidance on progression, and when to switch to other more challenging games and object manipulation tasks. In addition, two follow-up sessions were scheduled at 2 weeks and 6 weeks of the home program, where the exercises of the participants were re-evaluated and updated.

**Figure 4:**
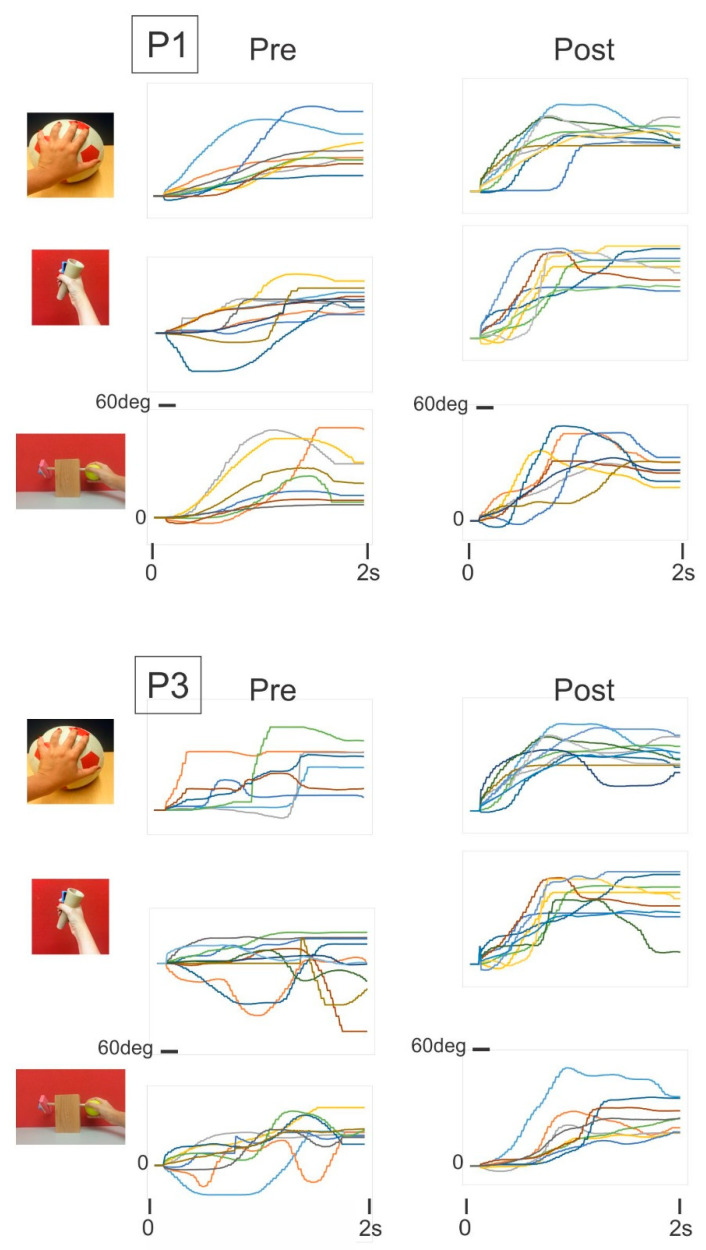
Game movement responses for three test object manipulation tasks of two children: pre- and post-intervention.

Pre- and post-intervention assessments included the following:

1. Two subtests of the PDMAS-2: (a) a 26-item grasping subtest, which measures a child’s ability to use their hands; (b) a 72-item VMI subtest, which measures a child’s ability to use visual perceptual skills. Both the grasp and VMI subtest scores have shown high test-retest reliability and have good construct validity [46];
2. The CUE assessment tool as described earlier and illustrated in Figure 4.

The following three object manipulation tasks were used to assess manual dexterity:

(i) Soccer ball: Children were instructed to place their open hand on top of the ball and roll it backward and forward to play the motor skill game module by using a combination of shoulder and elbow motion, and while maintaining finger extension;
(ii) Cone: Children grasped the cone with their whole hand and were asked to play the motor skill game module using pronation and supination;
(iii) 2-finger fine rotation: Children grasped the end of a small diameter wooden dowel between the tips of their thumb and index finger and were asked to play the motor skill game (MSG) module by rotating the wooden dowel leftward and rightward. Figure 3 presents a screenshot of the CUE assessment game and individual game movement trajectories by direction. The following outcome measures were derived from the recorded game movement responses of each test object manipulation task: success rate (SR), movement onset time (MOT), and magnitude of movement errors (ME).

All six children fully complied with the 12-week program, of four exercise sessions per week, each lasting at least 30 min. As presented in Table 2, there were modest improvements observed in the fine motor tasks of the PDMS-2. On average, the PDMS-2 grasp scores improved by 12% pre- to post-intervention and by 8% for the VMI scores. Figure 4 presents representative plots of game movement responses of three object manipulation tasks (CUE assessment) of two children pre- and post-intervention. On visual inspection of the game movement responses of one game session, improvement is evident in movement amplitude, movement quality, and movement variation. As presented in Table 3, Table 4 and Table 5, there were substantial improvements in the performance measures of the three CUE object manipulation tasks for all six children. On average there was a 30–55% improvement in success rate, and a 13–18% reduction in response times.

The preliminary findings of the two telerehabilitation pilot studies described above demonstrate feasible assessment and trial procedures, acceptable game-based, task-specific home training with a high compliance rate and positive outcomes. These findings and the theoretical evidence justify the next phase of a full-scale randomized controlled trial (RCT) for both adult stroke patients and children with CP.

## 10. Discussions

One limitation of the GTP is that it requires an IB computer mouse and a computer. Another limitation is that the IB mouse detects angular motion only. Therefore, it is not straightforward to practice tasks that require only linear motions. There are several fine motor skills that cannot be performed with the GTP approach using the IB mouse such as writing, doing up buttons, tying shoelaces or cutting food.

It was also realized that there are many reasons why patients may not adhere to a prescribed home exercise program and may not accept or be able to manage new rehabilitation technologies [17,18,19]. We expect that the features of the GTP and use of pervasive computer technologies, will provide new affordable eHealth solutions that will affect adherence to home exercise programs for people of all ages with chronic neurological disorders. A broad range of objects can be used to exercise two, three or four fingers or the whole hand/arm, tailored to individual needs. Many inexpensive commercial computer games can be used, which are engaging and require the goal-directed movement of increasing amplitude, speed, and accuracy. Knowledge of the therapeutic value (object and games activities) allow the therapists to prescribe an integrated program to target specific goals, for example, speed, accuracy, endurance, visuospatial functions, which are tailored to the individual child’s abilities.

The inclusion of a gaming element in the GRP is intended to provide extra motivation for children as a more enjoyable means of encouraging them to follow tedious, repetitive movements that are part of the rehabilitation process. Other studies that have incorporated computer games in therapy programs of the upper extremity have reported that patients enjoyed the games used in their exercise program [42,47,48]. In order to better understand the effectiveness of the GRP to increase the intrinsic and extrinsic level of motivation in children with CP and adult stroke patients, the theories of motivation, such as the achievement goal theory [49] can be used.

The presented preliminary results of a limited sample showed improvements in all six children in PDMS-2 grasp and visuomotor subtests, and improvements in performance metrics (success rate, movement onset time and magnitude of movement errors) of a broad range of precision, and goal-directed object manipulation tasks. The findings are consistent with previous studies that demonstrate that high repetition and task-specific training are effective in promoting the recovery of upper extremity function [10,13]. One main feature of the present game-assisted exercise program is to increase the number of repetitions of goal-directed movements (i.e., graded precision). Typically, each game-object combination is played for five minutes, and the duration of each game event is on average about two seconds. Thus, a 30-min exercise session would involve several game-object exercise trials and, several hundred game movement responses. This is a high number of repetitions of precision, goal-directed movements and of varying amplitude speed and direction. For most games that were used, the game targets appeared at random locations and therefore movement directions and amplitudes were varied. In addition, visual feedback is used to guide the object manipulations. This type of practice would promote implicit learning of eye-hand coordination [26].

## 11. Conclusions

Recovery programs can be extensive, for many children and adults with neurological disorders/injuries, including maintenance exercises that need to be performed continually to maintain functions and to prevent secondary disabilities. This paper introduced a game-based telerehabilitation platform (GTP) that provides, game-based exercise programs for recovery of manual dexterity and to automatically record and assess a patient’s fine and gross hand motor skills. The innovation of this approach comes from the implementation of affordable and easy-to-use objects, computer games and interfacing between the two. The GTP can initially be used in a supervised clinical setting, followed by a transition to function at home and can be monitored by clinician specialists. Clinical support of homes and remote communities with protocols that can be easily updated, will help increase the accessibility to targeted and personalized solutions for patients and achieve the desired training effect.

The present findings are positive and support further research and development. The long-term effects of GTP training on manual dexterity in children with CP will need to be confirmed in future randomized controlled (RCT) trials. In addition to measures of structure and function, future RCT should also include outcome measures such as health-related quality of life and level of participation to validate the findings. For children, outcome measures such as the goal attainment scale can be used [50]. For adult stroke patients, the stroke specific quality of life questionnaire can be used [51].

## Figures and Tables

**Table 1:** Demographic and clinical characteristics: Gross Motor Function Classification System (GMFCS) and Manual Ability Classification System (MACS).

Participants	Age (years)	Gender	GMFCS Level	MACS Level
P1	12	M	II	III
P2	10	M	I	II
P3	8	M	I	II
P4	7	F	II	III
P5	8	F	II	III
P6	5	M	II	III

**Table 2:** Pre- and post-exercise test scores and percentage change for PDMS-2 grasp and visuomotor subtests for each client.

	PDMS-2 Grasp			PDMS-2 Visuomotor		
	Pre	Post	% Change	Pre	Post	% Change
P1	46	52	13	110	1120	9
P2	47	52	11	120	130	8
p3	44	50	14	116	125	8
P4	46	51	11	120	131	9
P5	44	49	11	126	134	6
P6	44	50	14	113	127	12
Average			12			9

**Table 3:** Pre- and post-exercise test scores and percentage of change in soccer ball manipulation task for each child. Values are the average of forward and backward rolling game movements.

	Success Rate (%)			Response Time (ms)		
	Pre	Post	% Change	Pre	Post	% Change
p1	45	87	93	572	535	−6
p2	70	78	11	436	359	−18
p3	54	66	22	453	470	4
P4	93	93	0	570	527	−8
P5	30	84	180	732	527	−28
P6	55	100	82	687	532	−23
Average			65%			−13

**Table 4:** Pre- and post-exercise test scores and percentage of change in cone manipulation task for each child. Values are the average of pronation and supination game movements.

	Success Rate (%)			Response Time (ms)		
	Pre	Post	% Change	Pre	Post	% Change
p1	42	66	57	415	353	−16
p2	53	88	66	370	334	−10
p3	45	60	33	497	451	−9
P4	71	95	34	510	475	−7
P5	57	100	75	565	521	−8
P6	90	90	0	578	411	−29
Average			44%			−13

**Table 5:** Pre- and post-exercise test scores and percentage change in the fine rotation manipulation task for each child. Values are the average of leftward and rightward rotation game movements.

	Success Rate (%)			Response Time (ms)		
	Pre	Post	% Change	Pre	Post	% Change
P1	52	70	33	520	500	4
P2	70	95	34	665	440	34
P3	62	69	11	830	680	18
P4	45	45	80	540	460	18
P5	75	75	13	650	526	19
P6	74	80	9	625	552	12
Average			30%			18

## Data Availability

Data is available upon request.

## Appendix A

**Table A1:** Example computer games downloaded from www.Bigfishgames.com (accessed on 1 August 2021) and used for the experimental exercise programs. Note that matching and shooting games require children to utilize a small wireless optical computer mouse with a left-mouse button to press when needed.

Big Fish Game	Axis Play	Start Difficulty	Type	Clicker	Precision	Distractor	Executive Function
Action ball	Horizontal	Moderate	Brick buster	No	Moderate	Yes	Visual tracking and spatial
Aqua Ball	Horizontal	Easy	Brick buster	No	Low	Yes	Visual tracking and spatial
Astrobugs Revenge	Horizontal	Difficult	Match 3	Yes	High	No	Matching
Birds Town	Horizontal	Moderate	Match 3	Yes	High	No	Matching
Brave Piglet	Vertical	Moderate	Shooting	Yes	High	Yes	Visual tracking and spatial
Bricks of Egypt	Horizontal	Easy	Brick buster	No	Variable	Yes	Visual tracking and spatial
Butterfly Escape	Horizontal	Moderate	Match 3	Yes	High	Yes	Visual tracking and spatial
Bubble Town	Horizontal	High	Match 3	Yes	High	No	Matching, pairing
Chicken Invaders	Variable	High	Shooting	Yes	Moderate	Yes	Search and select
Clear It 2	Variable	High	Match 3	Yes	High	No	Visual tracking and spatial
Egyptian Ball	Horizontal	Difficult	Brick buster	No	Moderate	No	Search and select
Hyperballoid	Horizontal	Difficult	Brick buster	No	Moderate	No	Visual tracking and spatial
Invadazoid	Horizontal	Difficult	Brick buster	No	High	Yes	Visual tracking and spatial
Jar of Marbles	Horizontal	Easy	Match 3	Yes	Medium	No	Matching 3 aligning
Jet Jumper	Variable	Difficult	Driving Game	Yes	High	Yes	Visual tracking and driving
Luxor 3	Horizontal	Moderate	Match 3	Yes	High	Yes	Matching 3 aligning
Luxor HD	Horizontal	Moderate	Match 3	Yes	High	Yes	Matching 3 aligning
Luxor 5th Passage	Horizontal	Moderate	Match 3	Yes	High	Yes	Matching 3, aligning
Ozzy bubbles	Horizontal	Difficult	Aim and move	Yes	Low	Yes	Driving game
Ricochet Recharge	Horizontal	Moderate	Brick buster	No	High	No	Visual tracking and spatial

## References

[1] Cans C., Dolk H., Platt M., Colver A., Prasauskene A., Rägeloh-mann I.K. (2007). Recommendations from the SCPE collaborative group for defining and classifying cerebral palsy. Dev. Med. Child Neurol..

[2] James S., Ziviani J., Ware R.S., Boyd R.N. (2015). Relationships between activities of daily living, upper limb function and visual perception in children and adolescents with unilateral cerebral palsy. Dev. Med. Child Neurol..

[3] Di Carlo A. (2009). Human and economic burden of stroke. Age Ageing.

[4] Hendricks H.T., Van Limbeek J., Geurts A.C., Zwarts M.J. (2002). Motor recovery after stroke: A systematic review of the literature. Arch. Phys. Med. Rehabil..

[5] Mutai H., Furukawa T., Araki K., Misawa K., Hanihara T. (2013). Long-term outcome in stroke survivors after discharge from a convalescent rehabilitation ward. Psychiatry Clin. Neurosci..

[6] Hoare B.J., Wasiak J., Imms C., Carey L. (2007). Constraint-induced movement therapy in the treatment of the upper limb in children with hemiplegic cerebral palsy. Cochrane Database Syst. Rev..

[7] Klingels K., Feys H., Molenaers G., Van Daele S., Hoskens J., Desloovere K., De Cock P. (2013). Randomized trial of modified constraint-induced movement therapy with and without an intensive therapy program in children with unilateral cerebral palsy. Neurorehabil. Neural Repair.

[8] Etoom M., Hawamdeh M., Hawamdeh Z., Alwardat M., Giordani L., Bacciu S., Scarpini C., Foti C. (2016). Constraint-induced movement therapy as a rehabilitation intervention for upper extremity in stroke patients: Systematic review and meta-analysis. Int. J. Rehabil. Res..

[9] Hatem S.M., Saussez G., Della Faille M., Prist V., Zhang X., Dispa D., Bleyenheuft Y. (2016). Rehabilitation of motor function after stroke: A multiple systematic review focused on techniques to stimulate upper extremity recovery. Front. Hum. Neurosci..

[10] Gelkop N., Burshtein D.G., Lahav A., Brezner A., Al-Oraibi S., Ferre C.L., Gordon A.M. (2015). Efficacy of constraint-induced movement therapy and bimanual training in children with hemiplegic cerebral palsy in an educational setting. Phys. Occup. Ther. Pediatr..

[11] Bleyenheuft Y., Dricot L., Gilis N., Kuo H.C., Grandin C., Bleyenheuft C., Gordon A.M., Friel K.M. (2015). Capturing neuroplastic changes after bimanual intensive rehabilitation in children with unilateral spastic cerebral palsy: A combined DTI, TMS and fMRI pilot study. Res. Dev. Disabil..

[12] Muratori L.M., Lamberg E.M., Quinn L., Duff S.V. (2013). Applying principles of motor learning and control to upper extremity rehabilitation. J. Hand Ther..

[13] Maier M., Ballester B.R., Verschure P.F. (2019). Principles of neurorehabilitation after stroke based on motor learning and brain plasticity mechanisms. Front. Syst. Neurosci..

[14] Remple M.S., Bruneau R.M., Vanden Berg P.M., Goertzen C., Kleim J.A. (2001). Sensitivity of cortical movement representations to motor experience: Evidence that skill learning but not strength training induces cortical reorganization. Behav. Brain Res..

[15] Miller K.K., Porter R.E., DeBaun-Sprague E., Van Puymbroeck M., Schmid A.A. (2017). Exercise after stroke: Patient adherence and beliefs after discharge from rehabilitation. Top. Stroke Rehabil..

[16] Novak I. (2011). Effective home programme intervention for adults: A systematic review. Clin. Rehabil..

[17] Argent R., Daly A., Caulfield B. (2018). Patient involvement with home-based exercise programs: Can connected health interventions influence adherence?. JMIR Mhealth Uhealth.

[18] Jurkiewicz M.T., Marzolini S., Oh P. (2011). Adherence to a home-based exercise program for individuals after stroke. Top. Stroke Rehabil..

[19] Lin K.C., Wang T.N., Wu C.Y., Chen C.L., Chang K.C., Lin Y.C., Chen Y.J. (2011). Effects of home-based constraint-induced therapy versus dose-matched control intervention on functional outcomes and caregiver well-being in children with cerebral palsy. Res. Dev. Disabil..

[20] Beveridge B., Feltracco D., Struyf J., Strauss E., Dang S., Phelan S., Wright F.V., Gibson B.E. (2015). “You gotta try it all”: Parents’ Experiences with Robotic Gait Training for their Children with Cerebral Palsy. Phys. Occup. Ther. Pediatrics.

[21] Karamians R., Proffitt R., Kline D., Gauthier L.V. (2020). Effectiveness of virtual reality and gaming-based interventions for upper extremity rehabilitation poststroke: A meta-analysis. Arch. Phys. Med. Rehabil..

[22] Hickman R., Popescu L., Manzanares R., Morris B., Lee S.P., Dufek J.S. (2017). Use of active video gaming in children with neuromotor dysfunction: A systematic review. Dev. Med. Child Neurol..

[23] Chiu H.C., Ada L., Lee H.M. (2014). Upper limb training using Wii Sports ResortTM for children with hemiplegic cerebral palsy: A randomized, single-blind trial. Clin. Rehabil..

[24] Jannink M.J.A., Van Der Wilden G.J., Navis D.W., Visser G., Gussinklo J., Ijzerman M. (2008). A low-cost video game applied for training of upper extremity function in children with cerebral palsy: A pilot study. Cyberpsychol. Behav..

[25] Smeragliuolo A.H., Hill N.J., Disla L., Putrino D. (2016). Validation of the Leap Motion Controller using markered motion capture technology. J. Biomech..

[26] Panny M., Mayr A., Nagiller M., Kim Y. (2020). A domestic robotic rehabilitation device for assessment of wrist function for outpatients. J. Rehabil. Assist. Technol. Eng..

[27] Gerber C.N., Kunz B., Van Hedel H.J.A. (2016). Preparing a neuropediatric upper limb exergame rehabilitation system for home-use: A feasibility study. J. Neuroeng Rehabil..

[28] Sundaram S., Kellnhofer P., Li Y., Zhu J.Y., Torralba A., Matusik W. (2019). Learning the signatures of the human grasp using a scalable tactile glove. Nature.

[29] Miall R.C., Rosenthal O., Ørstavik K., Cole J.D., Sarlegna F.R. (2019). Loss of haptic feedback impairs control of hand posture: A study in chronically deafferented individuals when grasping and lifting objects. Exp. Brain Res..

[30] Burdea G.C., Grampurohit N., Kim N., Polistico K., Kadaru A., Pollack S., Oh-Park M., Barrett A.M., Kaplan E., Masmela J. (2020). Feasibility of integrative games and novel therapeutic game controller for telerehabilitation of individuals chronic post-stroke living in the community. Top. Stroke Rehabil..

[31] Aubin P.M., Sallum H., Walsh C., Stirling L., Correia A. (2013). A pediatric robotic thumb exoskeleton for at-home rehabilitation: The Isolated Orthosis for Thumb Actuation (IOTA). Proceedings of the 2013 IEEE International Conference on Rehabilitation Robotics (ICORR).

[32] Weightman A., Preston N., Levesley M., Holt R., Mon-Williams M., Clarke M., Cozens A.J., Bhakta B. (2011). Home based computer-assisted upper limb exercise for young children with cerebral palsy: A feasibility study investigating impact on motor control and functional outcome. J. Rehabil. Med..

[33] Posada-Gómez R., Montaño-Murillo R.A., Martínez-Sibaja A., Alor-Hernández G., Aguilar-Lasserre A.A., Reyes-Fernández M.C. (2018). An Interactive System for Fine Motor Rehabilitation. Rehabil. Nurs. Off. J. Assoc. Rehabil. Nurses.

[34] Bhattacharjya S., Stafford M.C., Cavuoto L.A., Yang Z., Song C., Subryan H., Xu W., Langan J. (2019). Harnessing smartphone technology and three dimensional printing to create a mobile rehabilitation system, mRehab: Assessment of usability and consistency in measurement. J. Neuroeng. Rehabil..

[35] Szturm T., Peters J.F., Otto C., Kapadia N., Desai A. (2008). Task-specific rehabilitation of finger-hand function using interactive computer gaming. Arch. Phys. Med. Rehabil..

[36] Szturm T., Sakhalkar V., Boreskie S., Marotta J.J., Wu C., Kanitkar A. (2015). Integrated testing of standing balance and cognition: Test-retest reliability and construct validity. Gait Posture.

[37] Kanitkar A., Szturm T., Parmar S., Gandhi D.B., Rempel G.R., Restall G., Sharma M., Narayan A., Pandian J., Naik N. (2017). The effectiveness of a computer game-based rehabilitation platform for children with cerebral palsy: Protocol for a randomized clinical trial. JMIR Res. Protoc..

[38] Lockery D., Peters J.F., Ramanna S., Shay B.L., Szturm T. (2011). Store-and-feedforward adaptive gaming system for hand-finger motion tracking in telerehabilitation. IEEE Trans. Inf. Technol. Biomed..

[39] Imran Z. (2017). Development of a Computerized Assessment Tool for Hand-Arm Function after Stroke-Test-Retest Reliability and Ronvergent Validity. MSc Thesis.

[40] Kanitkar A., Parmar S., Szturm T., Restall G., Rempel G., Naik N. (2021). Reliability and validity of a computer game-based tool of upper extremity assessment for object manipulation tasks in children with cerebral palsy. J. Rehabil. Assist. Technol. Eng..

[41] Kanitkar A. Repetitive Task Practice-Based Computer Game-Assisted Rehabilitation Platform for Hand and Arm Impairments in Children with Cerebral Palsy. Ph.D. Thesis.

[42] Kanitkar A., Parmar S.T., Szturm T.J., Restall G., Sepehri N. (2021). Parents’ perspectives on a computer game–assisted rehabilitation program for manual dexterity in children with cerebral palsy: Qualitative analysis of expectations, child engagement, and benefits. JMIR Rehabil. Assist. Technol..

[43] Mancini M.C., Brandão M.B., Dupin A., Drummond A.F., Chagas P.S.C., Assis M.G. (2013). How do children and caregivers perceive their experience of undergoing the CIMT protocol. Scand. J. Occup. Ther..

[44] Szturm T., Imran Z., Pooyania S., Kanitkar A., Mahana B. (2020). Evaluation of a game-based telerehabilitation platform for in-home therapy of hand-arm function poststroke: Feasibility study. PMR.

[45] Big Fish Games. https://www.bigfishgames.com/us/en.html.

[46] Van Hartingsveldt M.J., Cup E.H.C., Oostendorp R.A.B. (2005). Reliability and validity of the fine motor scale of the Peabody Developmental Motor Scales-2. Occup. Ther. Int..

[47] El-Shamy S.M. (2018). Efficacy of Armeo^®^ Robotic therapy versus conventional therapy on upper limb function in children with hemiplegic cerebral palsy. Am. J. Phys. Med. Rehabil..

[48] Ayed I., Ghazel A., Jaume-i-Capó A., Moyà-Alcover G., Varona J., Martínez-Bueso P. (2019). Vision-based serious games and virtual reality systems for motor rehabilitation: A review geared toward a research methodology. Int. J. Med. Inform..

[49] Palmer K.K., Chinn K.M., Robinson L.E. (2017). Using achievement goal theory in motor skill instruction: A systematic review. Sports Med..

[50] Steenbeek D., Ketelaar M., Galama K., Gorter J. (2007). Goal attainment scaling in paediatric rehabilitation: A critical review of the literature. Dev. Med. Child Neurol..

[51] Buck D., Jacoby A., Massey A., Ford G. (2000). Evaluation of Measures Used to Assess Quality of Life after Stroke. Stroke.

